# Sleeping With the Enemy? The Current Knowledge of Piscine Orthoreovirus (PRV) Immune Response Elicited to Counteract Infection

**DOI:** 10.3389/fimmu.2022.768621

**Published:** 2022-04-06

**Authors:** Eva Vallejos-Vidal, Felipe E. Reyes-López, Ana María Sandino, Mónica Imarai

**Affiliations:** ^1^ Centro de Biotecnología Acuícola, Facultad de Química y Biología, Universidad de Santiago de Chile, Santiago, Chile; ^2^ Facultad de Medicina Veterinaria y Agronomía, Universidad de Las Américas, Santiago, Chile; ^3^ Department of Cell Biology, Physiology, and Immunology, Universitat Autònoma de Barcelona, Barcelona, Spain; ^4^ Departamento de Biología, Facultad de Química y Biología, Universidad de Santiago de Chile, Santiago, Chile

**Keywords:** piscine orthoreovirus, double strand RNA (dsRNA) virus, heart and skeletal muscle inflammation (HSMI), antiviral immune response, pro-inflammatory cytokines, fish vaccines, aquaculture, emerging diseases

## Abstract

Piscine orthoreovirus (PRV) is a virus in the genus Orthoreovirus of the Reoviridae family, first described in 2010 associated with Heart and Skeletal Muscle Inflammation (HSMI) in Atlantic salmon (*Salmo salar*). Three phases of PRV infection have been described, the early entry and dissemination, the acute dissemination phase, and the persistence phase. Depending on the PRV genotype and the host, infection can last for life. Mechanisms of immune response to PRV infection have been just beginning to be studied and the knowledge in this matter is here revised. PRV induces a classical antiviral immune response in experimental infection of salmonid erythrocytes, including transcriptional upregulation of *ifn-α*, *rig-i*, *mx*, and *pkr*. In addition, transcript upregulation of *tcra, tcrb, cd2, il-2, cd4-1, ifn-γ, il-12, and il-18* has been observed in Atlantic salmon infected with PRV, indicating that PRV elicited a Th1 type response probably as a host defense strategy. The high expression levels of *cd8a*, *cd8b*, and *granzyme-A* in PRV-infected fish suggest a positive modulatory effect on the CTL-mediated immune response. This is consistent with PRV-dependent upregulation of the genes involved in antigen presentation, including MHC class I, transporters, and proteasome components. We also review the potential immune mechanisms associated with the persistence phenotype of PRV-infected fish and its consequence for the development of a secondary infection. In this scenario, the application of a vaccination strategy is an urgent and challenging task due to the emergence of this viral infection that threatens salmon farming.

## Introduction

Piscine orthoreovirus (PRV) is a virus that belongs to the *Reoviridae* family, *Spinareovirinae* subfamily, and genus *Orthoreovirus*. PRV was firstly described in 2010 (1), as it was associated with Heart and Skeletal Muscle Inflammation (HSMI) in Atlantic salmon (*Salmo salar*) (2). The disease was described in 1999 in fish farms in Norway (3). In Chile, the first report of PRV was published in 2016. Authors found PRV strains in HSMI lesions of farmed Atlantic salmon, and Coho salmon (*Oncorhynchus kisutch*) (4). HSMI has been diagnosed all the years over the last decade in more than 100 farm centers in Norway (excepting 2017 and 2019) (5), resulting in economic losses estimated at €9 million annually (6). In Chile, 3.7% of the infectious disease mortality of Atlantic salmon, and 14.9% of the infectious disease mortality of Coho salmon were associated with HSMI during 2020 (7). Because of the epidemiological evolution observed in the sanitary surveillance, HSMI was categorized as an emerging disease in Chilean salmon farming (7). The economic impact of PRV infection is associated with mortality and melanized spots in salmon filets (8) that correlate with a pro-inflammatory environment (9). Another concern is that its etiological agent, the PRV, is reportedly spreading from farmed to wild Atlantic salmon with yet undetermined impacts (10). In seawater Atlantic salmon farms, the infection prevalence can reach up to 97% (11), and the etiological agent, PRV, is also present in freshwater Atlantic salmon presmolts with high frequency, found in parr between 30 and 60 grams (4).

## PRV and Heart and Skeletal Muscle Inflammation

**Figure 1:**
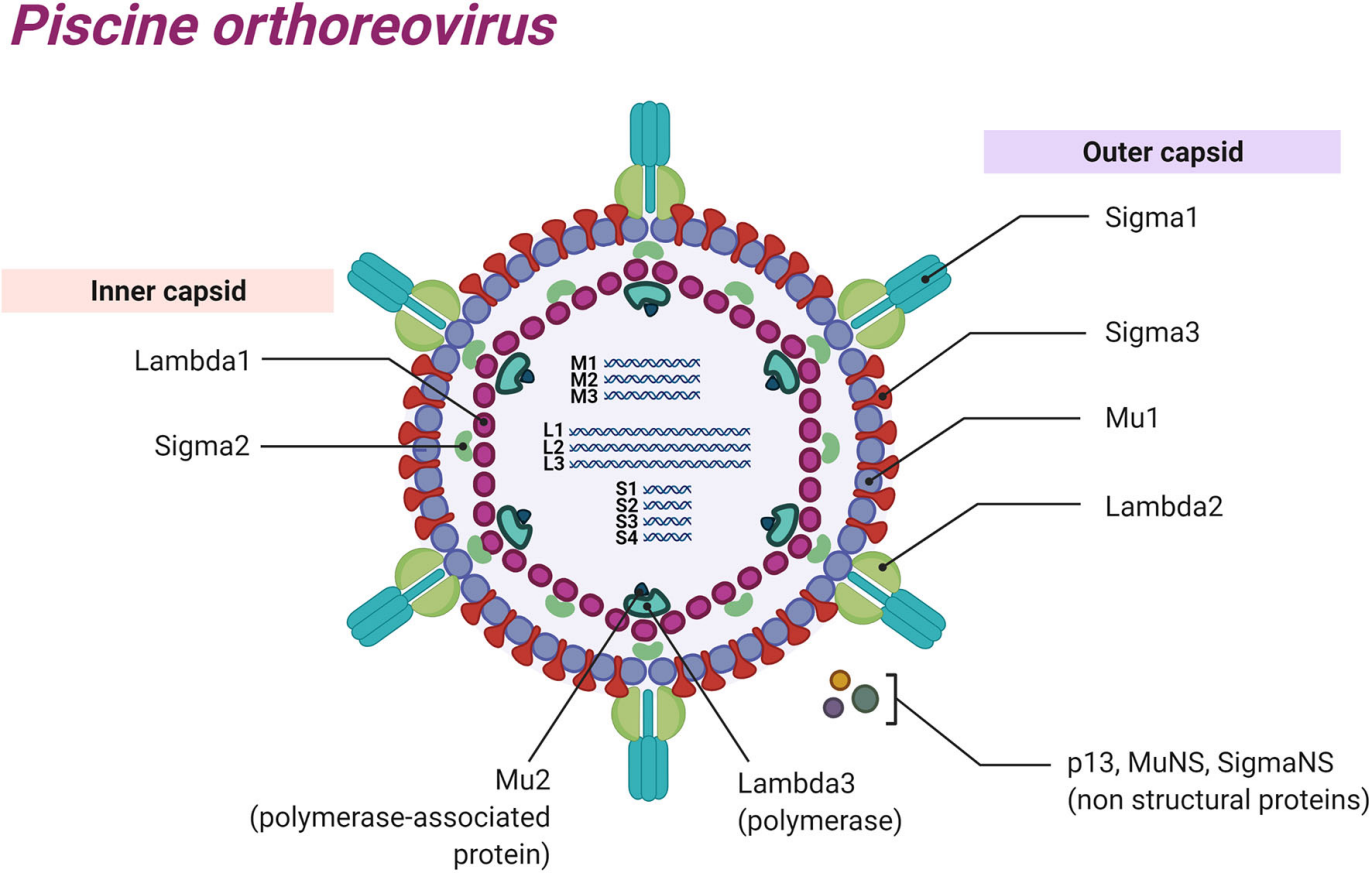
Schematic representation of Piscine orthoreovirus: Structural proteins, dsRNA segments and non-structural proteins are represented.

PRV is a dsRNA virus that has a genome composed of 10 RNA segments that can be classified into three different groups according to sizes: small segments (S1, S2, S3, S4) between 1040 and 1329 bp, medium (M1, M2, M3) between 2179 and 2403 bp, and large (L1, L2, L3) between 3911 and 3935 bp (1, 12, 13). The genome has at least 13 ORFs that encode for at least 11 proteins (14, 15). Eight of these proteins are structural components of the virus particle: segments L1, L3, M1, and S2 encode the inner capsid proteins λ1, λ3, µ2, and σ2, respectively; segments L2, M2, S1, and S4 encode for the outer capsid proteins λ2, µ1, σ3, and σ1, respectively; and segments S3, M3 and S1 encode for the three non-structural proteins σNS, µNS, and p13, respectively (**Figure 1**) (16, 17). PRV is a non-enveloped virus with an icosahedral structure (13).

Three different subtypes of PRV have been described using the coding sequence of PRV segments, denominated as PRV-1, PRV-2 and PRV-3 (18, 19). Phylogenetic analysis focused on the PRV genomic segments S1, differentiates this virus into two major genotypes, I and II, and each of them into two subgenotypes designated as Ia and Ib, and IIa and IIb, respectively. Subgenotypes Ia and Ib make up the PRV-1 subtype and subgenotypes IIb and IIa correspond to the PRV-2 and PRV-3 subtypes, respectively (20).

Recently, with all Gen Bank available PRV sequences (May 2020) and using new PRV S1 and M2 segment sequences was determined that a significant number of the publicly available sequences belong to the PRV-1 subtype (subgenotypes Ia and Ib), less belong to the PRV-3 subtype (subgenotype IIa) and there are few sequences of PRV-2 subtype (subgenotype IIb) (15). PRV is the etiological agent of HSMI in Norway, Canada, Germany, Scotland, Iceland, and Chile. Recently, it was suggested that PRV-1 subgenotype Ib can be responsible for HSMI in Atlantic salmon (15) while the subgenotype Ia was associated with low virulence (12, 15). PRV-2 is a virus found only in Coho salmon in Japan (not associated with HSMI symptoms); while PRV-3 induces a disease similar to HSMI in rainbow trout and salmon coho in Norway, Germany and Chile (12, 15, 21, 22).

Although it is necessary to complement the study using methodologies based on complete genome sequencing, besides segments S1 and M2 of PRV a lower resolution and representativeness of the remaining eight genomic segments for classifications of subgenotypes or subtypes have been observed. Phylogenetic trees support the original classification using the PRV genomic segment S1 (15).

Three phases have been described for PRV infection: *i)* the early entry and dissemination, *ii)* the acute phase and *iii)* persistence (14). Two to three weeks after the host entry, the replication and dissemination of the virus occurs into blood cells (23, 24). In this phase no infection *via* cohabitation has been described so far (24). To this date, there is no clarity about the mechanism of entry of the virus (14). The acute phase appears after 4 to 6 weeks of exposure to the virus and is characterized by the development of acute inflammation of the heart muscle and skeletal muscle, and substantial PRV replication in the erythrocyte occurs (14, 17, 24, 25). The duration of this phase depends on several factors related to the host immunity and on the PRV genotype causing the infection (26, 27). Cohabitation challenges show a successful infection at this point. This phase lasts between 1 to 2 weeks and then, the load of viral proteins drops dramatically in the erythrocytes, the clinical signs of the disease disappear, and the virus becomes persistent (17, 25). In the persistence phase, the viral RNA is found in erythroid progenitor cells, erythrocytes, macrophages, melano-macrophages, and other uncharacterized cells in the kidney (28). At this phase, poor viral infection is produced by cohabitation, but i.p. injected PRV inoculum prepared from clarified lysed blood cells from persistence phase Atlantic salmon accomplishes successful infection into naive fish (24). The extent of this phase depends on the PRV genotype and the host, but experimental trials have shown that this phase probably can last for life. The early entry and dissemination and acute phases have only been described under laboratory conditions.

Heart and skeletal muscle inflammation was first described in farmed Atlantic salmon in 1999 (3). The disease appears mainly between 5 to 9 months after salmon are transferred to marine water (2) but has also been described even earlier at 14 days of the seawater transfer (3). The clinical signs of the disease may emerge during the peak of the acute phase, but the clinical signs are found usually in the early stages of the persistent phase (24, 29, 30). In the field, the mortality of infected fish is usually low but can also go up to 20% of the infected cages (3). Macroscopic signs are pale heart, pericardial bleeding, ascites, and a pale or stained liver, but hematocrit levels are usually normal. Lesions can occasionally be found in the liver, spleen, gills, and kidney. The main histopathological lesions are in the heart and skeletal muscle. In the heart, necrosis of myocytes, infiltration with mononuclear cells (mainly lymphocytes and macrophages), and a massive inflammatory response are observed associated with myocardial degeneration (26). Pericarditis is usually found in association with myocarditis (2), also, perivasculitis is found in myocardial blood vessels, coronary veins, and grooves. In severe cases, an infiltrative pattern is also found (26). Red muscle inflammation follows the same pattern as seen in the heart but is not a consistent finding (2) and it has been proposed to be attributed to seasonal variation being most prevalent in autumn and winter (26). In experimental conditions, the production of cardiac lesions consistent with HSMI is related to the virulence of the isolate (24, 26, 31). At the same time, the highest virulent isolates correlated with higher plasma viremia, while the low virulent isolates showed a lower amount of virus detected by qPCR (31). There was no correlation between any specific viral gene, protein, or amino acid differences with the virulence of the distinct PRV-1 isolates analyzed although the virus strain and host specific factors are necessary to initiate HSMI (31). For example, in Canada, Pacific-adapted Mowi-McConnell Atlantic salmon infected with PRV shows only mild or non-heart inflammation, even though these fish show high blood viremia (24). A review of the biology, geographic distribution, and host range of PRV has been recently published and is recommended for deeper details of the knowledge on this virus and HSMI (14).

## Immune Response Against PRV Infection

PRV generates in *ex vivo* infected erythrocytes of Atlantic salmon upregulation of Interferon-a (*ifn-a)*, Retinoic acid-inducible gene I *(rig-i)*, Protein kinase R (*pkr)*, and Myxovirus resistance gene (*mx)*, all genes of the innate antiviral immune response (32, 33). In Atlantic salmon, intraperitoneal injected with PRV, *ifn-a* and *mx-α* were up-regulated in blood at 4 and 25 days post-challenge (dpc) and in heart, four dpc (34). The peak was in both tissues at four dpc, corresponding to the early infection stage, then the expression decreased to the control level (34). Similar results were reported in seawater adapted post-smolt salmon challenged with PRV by cohabitation. In these fish an increased expression of *ifn-a*, *rig-i, pkr, mx-α, viperin*, and *Interferon-stimulated gene 15 (isg15)* was observed in blood, heart, and in the spleen (35). In addition, a significant upregulation of both β-defensin and hepcidin genes in blood cells was reported at 4 weeks post challenge compared to day 0 (35). These studies show that PRV induces a strong innate immune response in Atlantic salmon, which may induce protection because the induced genes encode a dsRNA receptor like *rig-i* (35), interferon and interferon induced antiviral proteins such as *pkr, mx-α*, and *viperin*, and antiviral peptides like β-defensin and hepcidin (35). However, at this stage, it is unknown whether this response indeed results in protection because viruses have numerous evasion mechanisms against the IFN type I response (36). In fact, immune evasion mechanisms for IFN type I response have been reported for fish viruses such as IPNV, where preVP2, VP3, VP4, and VP5 viral proteins inhibit IFNa1 activation (37). Similarly, s7ORF1 of ISAV inhibits IFN and Mx transcription, while s8ORF2, acting as RNA silencing suppressor, inhibits IFN production (38–40). Therefore, further studies are required to understand whether PRV displays mechanisms to antagonize the IFN type I responses and antiviral peptides observed in infected fish.

Regarding adaptive immunity, transcript upregulation of T lymphocyte related genes such as T cell receptor-a (*tcra)*, *tcrb*, cluster of differentiation 2 (*cd2)*, and interleukin-2 (*il-2)* was reported in the head kidney of parr salmon challenged with PRV (41). In addition, *cd4-1*, the gene encoding the T cell co-receptor, was also upregulated in infected salmon (34), all indicating that PRV elicited the adaptive immune response in Atlantic salmon, which probably involves T cell proliferation. CD4^+^ CD3^+^ T cells (T helper) have been in fact identified, isolated and characterized in some fish species, including Japanese Pufferfish (*Takifugu rubripes*), ginbuna crucian carp (*Carassius auratus langsdorfii*), zebrafish (*Danio rerio*), rainbow trout (*Oncorhynchus mykiss*) (42–47); and rohu (*Labeo rohita*) (48). Although in Atlantic salmon, T cell isolation and characterization awaits further studies, transcriptional data and the studies in rainbow trout (44, 49–52) support the presence of T lymphocytes in this fish species and its role in response to the pathogens or model antigens. Furthermore, *ifn-γ* (41) and *il-12* were also upregulated in PRV infected salmon (34), indicating that a T helper type-1 response can take place as a host defense strategy. In teleost fish, the differentiation of naive CD4^+^ T cells into Th1 cells appears to be possible because these cells express both the *T-bet* master transcription factor and *ifn-γ* during differentiation (44, 45, 47). Other studies suggest that the differentiation of the CD4^+^ T cell into Th1, Th2, Th17, Treg lymphocytes can occur in fish, mostly based on the fact that many Th-type cytokine genes have been identified in fish (53) and are upregulated in lymphoid tissues and isolated T cells after antigen stimulation (53). A Th1 response could induce PRV clearance, as in the presence of IFN-γ and IL-12, cellular-mediated immune response can eradicate intracellular pathogens like viruses (54). Furthermore, in HSMI-sick Atlantic salmon hearts, a strong signal of MHC-II in the lesion areas and a moderate signal of CD3+ (55) by immunohistochemistry was detected, suggesting the activation of CD4+ T cells in response to PRV infection.

Following interaction with various bacterial and viral pathogens, macrophages become activated and secrete a wide range of antiviral, pro-inflammatory, and immunomodulatory cytokines (56). Mirroring the Th type differentiation, the macrophages are classified as M1-like or M2-like cells (57, 58) and they reflect the bidirectional macrophage–lymphocyte interaction. Using *in situ* hybridization to identify double MCSFR and PRV positive cells, it has been found that macrophage-like cells of spleen and kidney, and in melano-macrophages of kidney contain PRV-1, indicating that macrophages might be targets of infections (28). In PRV-infected salmon of a commercial farm, lesions of the white muscle, known as red spots, show abundant iNOS (inducible nitric oxide synthase) in positive M1-polarized macrophages infected with PRV-1. Transformation of red spots into black spots was associated with the presence of arginase-2 expressing M2 melano-macrophages and the reduction of the relative number of PRV-1 in the white skeletal muscle (9). Interestingly, in experimental PRV infection, M1 macrophages does not appear related to the infection damage of the heart in HSMI, although M2 macrophages in heart tissue suggested a role in HSMI recovery (59). Since PRV upregulates IFN-γ and IL-12 during viral infection of Atlantic salmon, it seems plausible that an efficient well-regulated response induces M1 macrophage differentiation for virus clearance in the heart. Furthermore, as IFN-γ can also increase CD80/86, CD83, and MHC-II levels in salmon immune cells, an efficient clearance can also be due to antigen presentation and recognition improvement leading to a rapid immune response (60).

Regarding cellular immunity, recent studies shed light on the potential role of the cytotoxic T lymphocytes (CTL) during PRV infection. First, the high expression levels of *cd8a, cd8b*, and *granzyme-A* in the head kidney of PRV-infected fish suggest a positive modulatory effect on the CTL-mediated immune response (34) because fish CTLs express CD8 co-receptor and enzymes able to induce apoptosis of the target cells (61). Recently, a study has shown that Atlantic salmon CD8+ cells appear abundant in areas of the heart that contain PRV-1 infected cells after experimental challenge. Moreover, upregulation of CD8α correlated in time with a moderate decline in PRV-1 RNA levels (59). Interestingly, these results suggest a role of CD8+ cells in virus clearance but direct evidence for the role of functional CTLs (CD8+ T cells) during PRV infection is not available yet. The evaluation of PRV-specific CTL function will require haplotype-matched between the effector and target cells, which can be achieved using clonal teleost fish (62, 63) or infected autologous cells (64, 65). The potential role of CTL in PRV infection of fish is consistent with the Th1 type response observed in salmon because IFN-γ can stimulate the development of CD8+ T cells during viral infection (66). It is also consistent with the fact that PRV infection of salmon erythrocytes induced upregulation of the genes involved in antigen presentation *via* MHC class I, including transporters like *tapasin* (*tapbp*) and proteasome components like *proteasome subunit beta type 9* (*psmb9a*) and *proteasome subunit beta type 6* (*psmb11*) (33), which will positively impact the activation of CD8+ T cells. Activating the antigen-presenting machinery may also be a consequence of IFN-γ upregulation during PRV infection because, in turn, this cytokine upregulates many genes involved in antigen presentation (67).

Finally, regarding humoral immune response against PRV, production of IgM against the PRV μ1 and μNS proteins has been detected in plasma of PRV-1-infected Atlantic Salmon (68). Using a bead based multiplex immunoassays, anti-s1 IgM was also detected in salmon seven weeks after the exposure of PRV shedders. A reduction in HSMI lesions was observed when the specific IgM production reached a maximum level, suggesting a protective effect, even though this humoral immune response has been insufficient to eradicate PRV as viral RNA persisted in the blood of fish with PRV-specific IgM in challenge trials (68). Similarly, in PRV-3-infected rainbow trout a significant increase in -specific IgM in plasma is reported 8 weeks after the exposure (29). Notably, no studies are measuring neutralizing antibodies.

**Figure 2:**
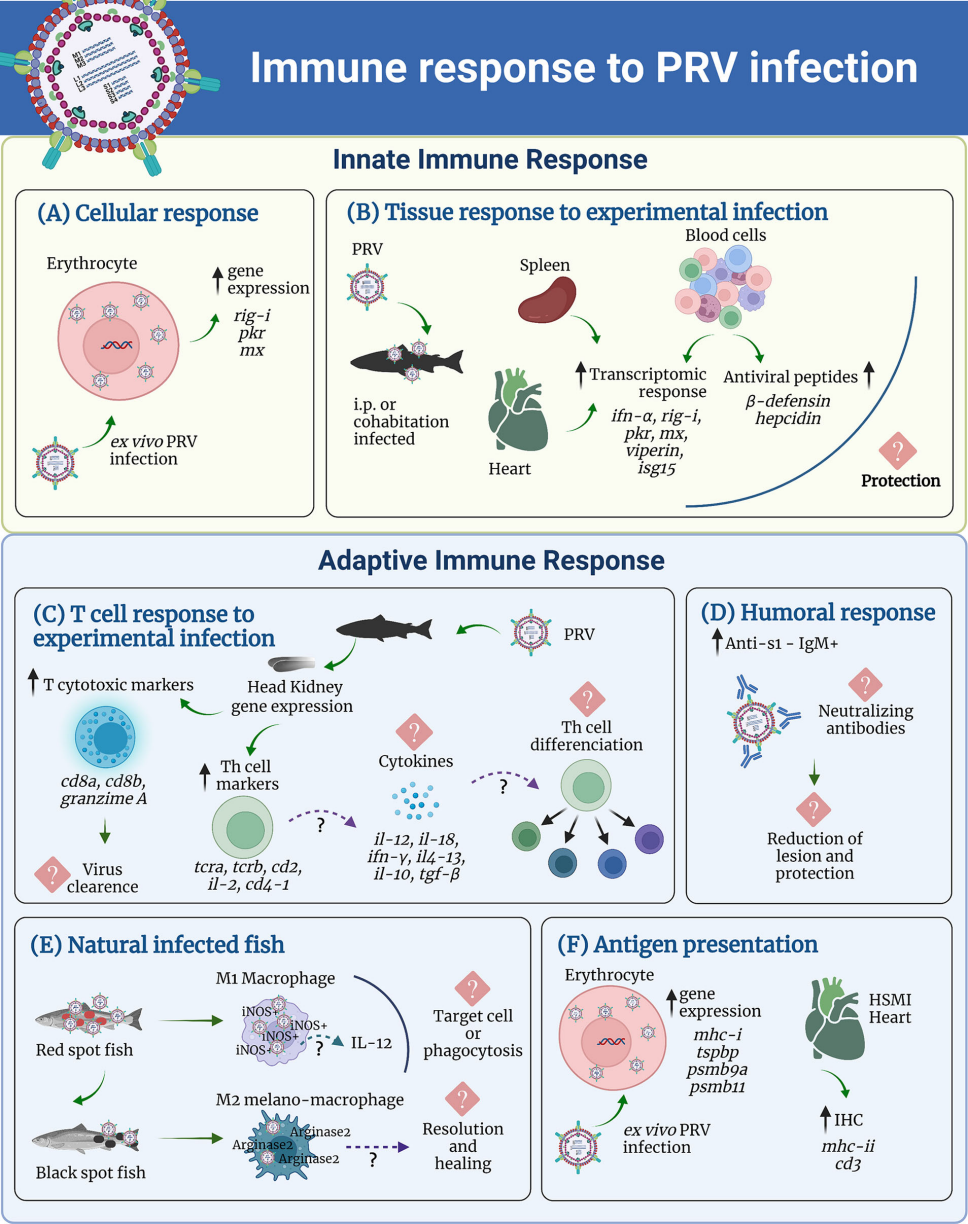
Summary of the current knowledge of the innate and adaptive immune response elicited by PRV infection. In the innate immune response panel, the transcriptome for **(A)** Cellular response, and **(B)** Tissue response to experimental infection is represented. For the adaptive immune response panel are shown, **(C)** T cell response to experimental infection, **(D)** humoral response, **(E)** macrophages response in naturally infected fish, and **(F)** Antigen presentation.

The current knowledge of the innate and adaptive immune response elicited by PRV infection is summarized in **Figure 2**.

## When the Fish Decides to Live With the Enemy: The Phenotype of Persistent Viral Infection in Atlantic Salmon

The persistence is a complex meta-stability exercise involving the overall outcome that favors the coexistence of the viral infection on the host, being considered one of the most successful surveillance strategies in the host-pathogen repertoire (69). In mammals, the phenotype of viral persistence is directly associated with the activity of inhibitory/immunosuppressive cytokines (70). In this process, the modulation of anti-inflammatory cytokines helps establish chronic viral infection (71–73). One of these anti-inflammatory cytokines of the viral persistence phenotype is IL-10, which impairs different immune mechanisms, including antigen recognition, cytokine production, antibody production, and cell proliferation processes, all vital processes for the success of immune response activation and resolution of infection (74). Consistently, viral persistence phenotype in Atlantic salmon infected with the infectious pancreatic necrosis virus (IPNV) is characterized by the upregulation of *il-10*, the low expression levels of *il-1b* and *il-8* and low levels of total IgM (75). In PRV-infected Atlantic salmon, the persistence phenotype has been recently reported (28). The description of this persistence phenotype is associated with a high level of viral RNA (28) and low levels of viral proteins in the erythrocytes (17), in which an antiviral innate immune response is observed at the transcriptional level (33). Not the cellular or molecular immune mechanisms responsible for or associated with the persistence phenotype of PRV-infected fish are known.

Thus, it seems reasonable that several regulatory mechanisms associated with the recognition and activation of the immune response are activated in the PRV persistence phenotype. They can contribute to establishing a weakened immune status, helping to develop secondary infections with highly prevalent pathogens in salmon farming. Only one study has evaluated the potential effect of PRV on the development of secondary infections, thus centering on the co-occurrence of PRV and salmonid alphavirus (SAV) (35). In co-infected Atlantic salmon (PRV- and then SAV-infected), lower SAV neutralizing titers were observed compared with the controls infected with SAV only (35) suggesting a detrimental effect for immunity. Moreover, a positive correlation between PRV and SAV was observed in moribund or dead salmon (35). These data suggest that PRV infection may affect the infection and susceptibility to other pathogens present in farmed fish. For instance, as far as we know, no articles are reporting the consequences of PRV on the development of secondary bacterial infections of high prevalence in salmon farming. Taking together, it is urgent from the sanitary point of view to elucidating the consequence of the high PRV prevalence upon the risk for the development of a secondary infection that the infection by PRV might produce by itself.

## PRV Vaccines

At present, there are no commercially available vaccines against HSMI in the market, and research and evaluation of PRV vaccines are still incipient. Only four studies performed on Atlantic salmon and Coho salmon have been reported (76–79). Wessel et al. (76) evaluated the protective effect of an inactivated vaccine using PRV purified from infected erythrocytes in a vaccination trial against HSMI.

Those immunized fish challenged with PRV by intraperitoneal injection showed a lower PRV load in blood cells and plasma compared both to PBS control group and vaccine control group (vaccinated with ALPHA JECT micro-6; PHARMAQ AS). Differences were observed for all the time-points assessed, i.e., 2-, 4-, 7-, and 10-weeks post-challenge (wpc). However, the PRV load was estimated based on the quantification cycle (Cq) value instead of absolute quantification. Beyond this methodological concern, only differences at 4wpc were registered between the PRV-inactivated vaccine group and the PBS-vaccinated group (76). Furthermore, the same trend was observed from the histopathological evidence on the heart. Thus, data indicate that PRV vaccination substantially reduced the severity of HSMI specific lesions, mainly in the experimental group following an unnatural way of infection (i.e., following an intraperitoneal injection), but did not prevent PRV infection and virus replication. Notably, a different study reported that the inactivated PRV-1 vaccine does not prevent PRV-1 infection and only partially protects against HSMI (78). Regrettably, the applicability of these vaccines is minimal because there are currently no reports of a cell line capable of producing PRV-1 viral progeny (80).

One common and ancient strategy for successful immunization is the cross-protection induced by related low virulent virus variants to cause low-grade disease (78). Particularly for HSMI, the cross-protection has been assessed using PRV-2 and PRV-3 genotypes not associated with disease development in Atlantic salmon. The cross-protection assay showed that the primary infection with intraperitoneally injected PRV-3 genotype completely blocked the infection against PRV-1 and the development of HSMI in Atlantic salmon ten weeks later the immunization with PRV-3 (78), which is in agreement with the fact that PRV-3 induces a disease similar to HSMI in rainbow trout and salmon coho (12, 15, 18, 21, 22, 78). The mechanisms of protection induced by PRV-3 are not known. In fact, the gene expression analysis of cellular immunity indicators (*cd8α*, *ifn-γ*, and *granzyme-a*) indicated that PRV-3 did not trigger spleen upregulation of these genes beyond ten weeks (78). The authors also state that antiviral immune genes *viperin, myxovirus resistance gene* (Mx), and *interferon-stimulated gene* (ISG-15) did not change their expression pattern (78).

By contrast, PRV-2 infection did not prevent PRV-1 infection, reducing only the severity of HSMI pathology punctually in some few individuals (78). Since PRV-2 is the etiological agent of a different disease in coho salmon (*Oncorhynchus kisutch*), named erythrocytic inclusion body syndrome (EIBS) (18), this results may have been expected. Perhaps the protection is associated with the higher amino acid identity of PRV-1 with PRV-3 (90%) than PRV-2 (80%) (81). Importantly, the high identity between PRV-3 and PRV-1 is present in proteins probably involved in the pathogenic effects (82, 83), such as the outer clamp protein σ3 (79.1%) and the non-structural protein p13 (78.2%) (81). Beyond these unknowns, one critical concern on the use of PRV-3 in immunizing Atlantic salmon is the possibility that the RNA segmented of PRV-3 and PRV-1 could reassort if they infect the same cell (84), in which case the consequences are unpredictable. Therefore, the side-effects and potential consequences of this type of immunizing strategy must be carefully analyzed.

In aquaculture, there are DNA vaccines licensed for commercial use for protecting Atlantic salmon against viruses including Infectious Hematopoietic Necrosis Virus (IHNV) (APEX-IHN; Novartis/Elanco) (85), and Salmon Pancreas Disease Virus (SPDV) (86). In this scenario, the vaccine efficacy against HSMI following intramuscular-injected immunization was assessed using pSAV-based replicon vaccines and pcDNA3.1-based expression vaccines. The Atlantic salmon vaccinated with pcDNA3.1 vector expressing µNS, σNS, and σ1 controlled by a CMV promoter showed a substantial reduction in the viral RNA load and the HSMI histopathological changes in epicardium and ventricle (77). By contrast, the pSAV cocktail replicons containing µNS + µ1 + σNS + σ1 + σ3 + λ2, slightly reduced the cardiac histopathological score, but did not reduce the PRV RNA levels in the blood after infection compared to the control, suggesting that the type and number of different expression vectors may influence on such differences (77). The secretion of specific antibodies as an inducer of protection through DNA vaccines (87) was not evaluated. Based on the study of Haatveit et al. (77) it seems that µNS and σ1 are the most promising PRV antigens for a DNA vaccine against HSMI in Atlantic salmon. However, the mechanism of action of these proteins activating the immune response remains to be elucidated. Consequently, the application of DNA vaccine as prophylactic treatment against aquaculture-related viruses, including PRV, must be further assessed.

## Conclusions and Perspective

This article revised the current knowledge regarding the immune mechanisms activated in response to PVR infection. There is evidence that PRV activates the antiviral immunity in salmonid erythrocytes, one of its cellular infection targets. Currently, it is unclear whether activating an antiviral environment is enough to induce host protection. Because viruses, including aquatic viruses, show numerous immune evasion mechanisms against the IFN type I host response, understanding PRV-host interaction, which may antagonize the IFN type I response, needs to be addressed. The scope of other immune mechanisms against the PRV and its actual contribution to the resolution of infection, including the role of neutralizing antibodies, still need to be elucidated. Nonetheless, the eradication of virus does not always occur during infection in nature. In fact, for PRV, there are reports of persistent infection promoting host-pathogen coexistence. PRV persistence is characterized by immune modulators´ upregulation mainly associated with anti-inflammatory molecules. Consequently, this lower immune capacity to respond against a threat aggregates complexity to the mechanisms developed by the host for ensuring survival. Importantly, as far as we know, there is a gap in the knowledge concerning the cellular and molecular mechanisms responsible for the promotion of the persistence phenotype on PRV-infected fish.

The generation of further knowledge to understand the immune mechanisms in response to and for protection against PRV will make possible the development of strategies capable of effectively and efficiently facing this viral infection and the negative impact produced in the fish farming industry and the environment. Within the sustainable aquaculture industry framework, all the above is committed to the environment.

## Author Contributions

All the authors wrote, read and approved the original draft.

## Funding

This work has been supported by DICYT-USACH Postdoctoral fellowship (Nb. 022043IB) and Fondecyt iniciación (Proyect number 11221308).

## Conflict of Interest

The authors declare that the research was conducted in the absence of any commercial or financial relationships that could be construed as a potential conflict of interest.

## Publisher’s Note

All claims expressed in this article are solely those of the authors and do not necessarily represent those of their affiliated organizations, or those of the publisher, the editors and the reviewers. Any product that may be evaluated in this article, or claim that may be made by its manufacturer, is not guaranteed or endorsed by the publisher.
